# Secretory Phospholipase A2s in Insulin Resistance and Metabolism

**DOI:** 10.3389/fendo.2021.732726

**Published:** 2021-08-27

**Authors:** Michael S. Kuefner

**Affiliations:** Department of Molecular Medicine and Surgery, Karolinska Institutet, Stockholm, Sweden

**Keywords:** phospholipase A2, metabolism and obesity, Type 2 diabetes, insulin resistance, glucose homeostasis, lipid metabolism

## Abstract

The phospholipases A_2_ (PLA_2_) superfamily encompasses enzymes commonly found in mammalian tissues and snake venom. Many of these enzymes have unique tissue distribution, function, and substrate specificity suggesting distinct biological roles. In the past, much of the research on secretory PLA_2_s has analyzed their roles in inflammation, anti-bacterial actions, and atherosclerosis. In recent studies utilizing a variety of mouse models, pancreatic islets, and clinical trials, a role for many of these enzymes in the control of metabolism and insulin action has been revealed. In this review, this research, and the unique contributions of the PLA_2_ enzymes in insulin resistance and metabolism.

## Introduction

Metabolic syndrome constitutes an array of pathophysiologies, including obesity, glucose intolerance, and dyslipidemia. The most common feature between these pathophysiologies is insulin resistance, a condition in which cells fail to respond normally to insulin. One factor associated with insulin resistance is triacylglycerol accumulation in muscle, liver, and fat (1, 2). While triacylglycerols are a marker for insulin resistance (3), they may not be causal. Instead, cellular lipids with signaling roles (diacylglycerols, fatty acids, phospholipids, etc.) may fill this role, as accumulation of a variety of lipid species can cause insulin resistance (4–6).

One group of enzymes that produce these lipids are secretory phospholipases A_2_ (sPLA_2_s), a protein family found in mammalian tissues and snake venom which hydrolyze glycerophospholipid *sn-2* ester bonds, generating a non-esterified free fatty acid and a lysophospholipid. Secretory phospholipases A_2_s are low molecular weight enzymes (~14 kDa), most of which require millimolar amounts of Ca^2+^ to function. Twelve sPLA_2_ isoforms have been identified thus far, of which ten are catalytically active (IB, IIA, IIC, IID, IIE, IIF, III, V, X, XIIA), and two are dormant (XIIB and otoconin-90) (7, 8). These enzymes have varied expression patterns and substrate preferences, signifying diversity in their biological roles. Over the past couple decades of research, the majority of studies concerning sPLA_2_s dealt with their roles in cardiovascular disease, inflammation, antimicrobial actions, and membrane remodeling. However, a discussion on how sPLA2s may regulate or impact glucose metabolism, insulin signaling, and metabolism is lacking. Recent research has identified that at least 7 of these sPLA_2_s modulate glucose metabolism, presumably by generation of fatty acids or lipoproteins that influence lipid metabolism and mobilization, alterations in fatty acid oxidation, or other mechanisms involved in insulin signaling and obesity (**Table 1**).

**Table 1:** Metabolic roles of sPLA2 isoforms.

sPLA_2_ Isoforms	Primary Localization	Metabolic Implications	Reference
PLA2G1B	Pancreas, lung	Promotes weight gain; increases TG and cholesterol levels through elevated LPC intestinal absorption	(9–16)
PLA2G2A	Platelets, liver, leukocytes, paneth cells, adipose tissue	Controversial; promotes weight gain, insulin resistance in rats. Improves metabolic parameters in mice.	(17–21)
PLA2G2D	Lymph tissue dendritic cells	Undocumented; May be metabolically beneficial due to release of anti-inflammatory FAs/lipid mediators	(22–25)
PLA2G2E	Adipose	Controversial; *Pla2g2e^-/-^* male mice display blunted lipolysis and elevated TG storage. Other experiments using *Pla2g2e^-/-^* female mice found reduced lipid accumulation.	(26–29)
PLA2G5	Adipose, bronchial epithelial cells, hepatocytes, islets, macrophages, cardiomyocytes	Protective of diet-induced obesity and insulin resistance; pushes adipose tissue macrophages from M1➔M2 state.	(26)
PLA2G10	Lung, adrenal gland, brain, heart, adipose	Protective of diet-induced obesity. Improves TG clearance in adipose and suppresses glucocorticoid production in adrenal cells	(27, 30–34)
PLA2G12B	Liver, small intestine, kidneys	Strong regulation over hepatic lipoprotein packaging and VLDL secretion; expression protects from hepatosteatosis	(8, 35–45)

Tissue specific expression patterns and metabolic roles for the secretory phospholipases A2.

## PLA2G1B

The metabolic role of PLA2G1B has been clarified with *Pla2g1b* knockout (*Pla2g1b^-/-^*) mice or pancreatic acinar cell-specific PLA2G1B overexpression. PLA2G1B is mainly expressed in pancreatic acinar cells and the lungs, and only displays its enzymatic activity after feeding, as this causes the enzyme’s release into the pancreatic fluid and subsequent secretion into the intestinal lumen where it is proteolytically cleaved from an inactive state to its active form (46). Activated PLA2G1B contributes to lipid metabolism and absorption of lysophospholipids, particularly lysophosphatidylcholine (LPC). *Pla2g1b^-/-^* mice on a C57BL/6 background fed a hypercaloric diet (58.5% fat, 25% sucrose, 16.5% protein) for either 3 or 10 weeks are resistant to diet-induced obesity (9). *Pla2g1b^-/-^* mice showed a 37% reduction in plasma triglyceride (TG) levels primarily due to a decrease in hepatic VLDL production and an increase in TG-rich lipoprotein clearance. *Pla2g1b^-/-^* mice also displayed a 61% reduction in plasma cholesterol following 10 weeks on the hypercaloric diet compared to age-matched wild-type controls. Notably, *Pla2g1b^-/-^ Ldlr^-/-^* mice fed the same hypercaloric diet for 10 weeks displayed a similar phenotype, including reductions in fasting glucose, insulin, and plasma lipids (10). Similarly, wild-type mice consuming a high-fat, high-carbohydrate diet supplemented with the general sPLA_2_ inhibitor, methyl indoxam, showed a reduction in body weight after 10 weeks (11). This decrease in body weight was accompanied by enhanced glucose tolerance and suppression of post-prandial plasma lysophospholipid levels. Transgenic mice over-expressing the human *PLA2G1B* in pancreatic acinar cells gained more weight when given the hypercaloric high-fat/high-carb diet, and these mice also had reduced glucose tolerance and insulin resistance (12).

Given the strong evidence supporting PLA2G1B inhibition as an avenue for improving metabolic health, a discussion on the mode of action is warranted. Absorption of LPC into the portal blood, plasma, and livers was reduced in *Pla2g1b^-/-^* mice fasted for 12 hours followed by a glucose-lipid mixed meal (13). These data suggest that phospholipid digestion in the intestinal lumen and absorption of the digested lysophospholipid product through the portal blood is caused directly by PLA2G1B enzymatic activity following a meal (13). While PLA2G1B is the major enzyme for phospholipid hydrolysis within the intestinal lumen, other lipolytic enzymes may compensate in its absence to preserve lipid and cholesterol absorption (14). In regards to the enhanced glucose tolerance in *Pla2g1b^-/-^* mice, there is evidence that LPC alone has an adverse effect on hyperglycemia as shown with a glucose tolerance test (GTT) (13). Furthermore, *Pla2g1b^-/-^* mice have elevated fatty acid oxidation which can be directly suppressed by LPC, suggesting PLA2G1B enzymatic products reduce fatty acid oxidation (15).

Taken together, studies on the metabolic impact of PLA2G1B inhibition indicate it has metabolic effects in response to feeding. Recent experiments provide evidence that the benefits of PLA2G1B inhibition mimic those seen in response to bariatric surgery in mice, including prevention of dyslipidemia, and protection and remission from diet-induced Type 2 diabetes (16).

## PLA2G2A

PLA2G2A is induced by several cytokines and second messengers including interleukins 1 and 6 (IL-1 and IL-6), tumor necrosis factor (TNFα), lipopolysaccharides (LPS), and cyclic AMP, suggesting a pro-inflammatory role (47–49). The Pla2g2a knockout (*Pla2g2a^-/-^*) BALB/c mice have less joint inflammation than their wild-type counterparts under inflammatory conditions (50). With the focus of PLA2G2A studies being on its role in inflammation and atherosclerosis, studies on the actions of PLA2G2A in metabolism are quite limited. One consistent observation of the metabolic studies involving PLA2G2A is that its expression is up-regulated in response to a high-fat diet (17–19). In male Wistar rats, *Pla2g2a* expression was elevated 6-fold after 16 weeks on a high-carbohydrate high-fat (HCHF) diet (17). At 8 weeks of age, these rats were orally administered the PLA2G2A inhibitor KH064, which drastically reduced weight gain, fat mass, and prevented an increase of adipocyte crown formation and macrophage infiltration seen in the wild-type rats (17). Inhibition of Pla2g2a by KH064 was also accompanied by an increase in lipolytic gene expression, attributed to an increase in hormone-sensitive lipase (HSL) phosphorylation. Lastly, treatment with KH064 improved glucose tolerance and insulin sensitivity as assessed by GTTs and ITTs (17).

The metabolic phenotype of male C57BL/6 mice genetically engineered to overexpress the human *PLA2G2A* gene has also been assessed (51). PLA2G2A overexpression improved glucose clearance and insulin sensitivity in GTTs and ITTs, thereby alleviating obesogenic symptoms in response to HFD (18, 20). C57BL/6 mice normally do not express the murine *Pla2g2a* due to a frameshift mutation in exon 3 (52). Overexpression of human *PLA2G2A* protected mice from weight gain on a high-fat diet compared to wild-type C57BL/6 mice after 10 weeks. *PLA2G2A* expression also enhanced oxygen consumption (VO_2_) and energy expenditure. The expression of thermogenic genes in brown adipose tissue (BAT), including uncoupling protein 1 (UCP1), peroxisome proliferator-activated receptor γ coactivator 1α (PGC-1α), and Sirtuin-1 (SIRT1) was elevated (18, 20). *PLA2G2A-*expressing primary adipocytes from epididymal and inguinal white adipose tissue (WAT), and interscapular BAT showed elevated abundance of several proteins involved in adaptive thermogenesis compared to C57BL/6 wild-type adipocytes (20). To accompany this phenotype, mice expressing *PLA2G2A* also had reduced 6-hour fasting blood glucose levels and an increase in glucose transporter type 4 (GLUT4) in BAT suggesting that *PLA2G2A* enhances BAT glucose utilization (20).

PLA2G2A contributes to the inflammatory response, but the enzyme’s role in obesity and metabolism is still unclear. Contributing to this, current investigations of PLA2G2As metabolic role used different designs. The human PLA2G2A gene was expressed in mice, whereas the rat Pla2g2a enzyme was inhibited pharmacologically (17, 51). Overexpression of *PLA2G2A* in mice may alter the expression of other secretory phospholipases in a variety of tissues, which could influence metabolism. Similarly, the activity of various sPLA_2_ isozymes has not been examined in response to PLA2G2A inhibition by KH064. Moreover, food intake following KH064 administration was not reported in this study, and the impact of KH064 on intestinal lipid absorption was not assessed.

## PLA2G2E

The expression of PLA2G2E is elevated in the white adipose tissue (WAT) and BAT of female C57BL/6 mice fed a HFD (26). Conversely, female *Pla2g2e^-/-^* mice gain less weight on a HFD over 18 weeks, with marked reductions in fat mass, hepatic lipid deposition, plasma aspartate aminotransferase (AST), and alanine aminotransferase (ALT) levels. Thus, the level of PLA2G2E may affect adiposity and liver metabolism. PLA2G2E preferentially hydrolyzes PE and PS on very low-density lipoprotein (VLDL), low-density lipoprotein (LDL), and high-density lipoprotein (HDL), although with weak enzymatic activity compared to other sPLA_2_s (27, 28). Mass spectrometry of the lipoprotein particles from *Pla2g2e^-/-^* mice revealed a reduction in phospholipids (PL), triglyceride (TG), and cholesterol accumulation in VLDL, LDL, and HDL (26). These data suggest that Pla2g2e may promote obesity through elevated hepatic lipogenesis and VLDL assembly in the liver. Anionic phospholipids decrease the affinity for ApoE to bind the LDL receptor (LDL-r), which could impact lipoprotein particle clearance (53). Finally, whether lysophosphatidylserine (LPS) or lysophosphatidylethanolamine (LPE) impacts metabolism has yet to be determined.

In contrast, another study using male *Pla2g2e^-/-^* mice discovered the enzyme regulates lipolysis in adipocytes, likely through enhanced ERK1/2 signaling (29). Pla2g2e^-/-^ mice had increased epididymal fat compared to C57BL/6 wild-type mice and accumulated more TG in the SVF isolated from adipose tissue. Over-expression of Pla2g2e in OP9 stem cells or treatment of 3T3-L1 cells with Pla2g2e protein reduced lipid accumulation and increased release of free glycerol, indicative of elevated lipolysis (29). These *Pla2g2e^-/-^* animals had reduced ERK1/2 signaling and HSL, the intracellular lipase responsible for hydrolyzing TG to FFAs. Finally, treatment of adipocytes with mouse Pla2g2e protein induced ERK1/2 signaling, demonstrating that Pla2g2e regulates adipocyte lipolysis through ERK/HSL signaling (29).

Currently, the understanding of the role of PLA2G2E in obesity and metabolism is limited to the two studies described above which report contrasting phenotypes with *Pla2g2e^-/-^* mice. A notable difference between these studies is the use of females *versus* males, and the contrasting results suggest sex-specific differences in Pla2g2e activity and its impact on obesity may be involved.

## PLA2G5

PLA2G5 is mainly expressed in WAT and protects from diet-induced obesity. PLA2G5 expression is elevated in response to HFD feeding in female C57BL/6 mice (26). When placed on a HFD, *Pla2g5^-/-^* mice gained a large amount of weight, mainly from increased visceral fat mass (26). In GTTs and ITTs, the *Pla2g5^-/-^* mice had impaired glucose tolerance and insulin resistance. Furthermore, there was a striking induction of plasma ALT levels and hepatic fat deposition, indicating exacerbated hepatosteatosis (26). PLA2G5 preferentially hydrolyzes phosphatidylcholine (PC) in low-density lipoprotein (LDL) (54). In the *Pla2g5^-/-^* mice phospholipid, cholesterol, and TG levels were considerably higher in LDL (26). When transgenic mice overexpressing PLA2G5 in adipocytes were put on HFD, they showed better insulin sensitivity and a decreased expression of pro-inflammatory genes in WAT (26). These data suggest PLA2G5-mediated hydrolysis of PC and other phospholipids may reduce local adipose tissue inflammation which appears to have a beneficial impact on whole-body insulin sensitivity.

PLA2G5 also modulates bone marrow-derived macrophage (BMDM) polarization. BMDM treated with palmitic acid (PA) or lipopolysaccharides induces the inflammatory response. Addition of recombinant PLA2G5 enzyme augments the expression of M2 markers *Arg1* and *Cd206* in the BMDM, suggesting that PLA2G5 has anti-inflammatory effects (26). The capacity for PLA2G5 to push macrophage polarization from an M1 to M2 state broadens its impact as the metabolic benefits of M2 macrophages are well documented (55–57). Genetic deletion of Th2 or M2 inducers increases the risk for metabolic disorders (56), and M2 macrophage infusion into obese mice has proven to be effective in treating obesity and improving insulin sensitivity (57). In humans, M2 macrophages are more prevalent in adipose tissue from lean individuals (55). While additional work needs to be done to elucidate the effects of PLA2G5 on metabolism, these actions appear to be partly mediated by the fatty acid and/or PC-released induction of M2 macrophage polarization in adipose tissue.

The role of PLA2G5 in glucose-stimulated insulin secretion (GSIS) is complex. GSIS is decreased in isolated pancreatic islets from PLA2G5 knockout mice and in pancreatic MIN6 cells following siRNA-mediated PLA2G5 knockdown (58). Additionally, PLA2G5 overexpression in MIN6 cells enhanced GSIS and increased AA release into the media with no change in prostaglandin E2 (PGE2) abundance (58). In contrast to the studies with isolated islets, the GSIS of Pla2g5^-/-^ mice was increased compared to WT mice (58). The elevated GSIS was attributed to increased pancreatic islet size and number of proliferating cells in the pancreatic β-islets of the Pla2g5 KO mice. The *in vivo* data from this study suggests a reduction in the release of AA, a fatty acid contained in over 30% of glycerolipids in rodent islets (59), is beneficial for insulin secretion and β-cell proliferation. Amino acids generally induce GSIS, while inhibition of the release of AA inhibits GSIS (60). Paradoxically, a major metabolite of AA is PGE2, a well-known inhibitor of GSIS (61–63). The role of AA metabolites in GSIS will be discussed further in the next section regarding Pla2g10. The data suggest that PLA2G5 regulates insulin secretion and β-cell proliferation, which may be dependent on the amount of AA released *versus* the amount of AA used for PGE2 production.

## PLA2G10

Studies with transgenic mice provided evidence that PLA2G10 mediates adipogenesis and has thereby led to the hypothesis that it protects from diet-induced obesity (30, 31). PLA2G10 binds to zwitterionic phospholipids such as PC with high affinity, releasing AA and LPC. PLA2G10 is expressed in a variety of tissues including the lungs, adrenal glands, pancreas, brain, heart, and adipose tissue (27, 32–34). *Pla2g10^-/-^* mice on a C57BL/6 background gain more weight and adipose mass over 40 weeks compared to chow-fed wild-type mice (30). The effects of Pla2g10 deletion are directly on WAT, as there were no changes in food intake or respiration. Pre-adipocytes prepared from WAT of *Pla2g10^-/-^* mice accumulated more TG when induced to differentiate *ex vivo*, suggesting that PLA2G10 has a direct effect in adipose tissue to reduce lipid accumulation. Similarly, when the OP9 cell line was modified to overexpress PLA2G10, TG accumulation was reduced following differentiation (30). In addition, the cells had reduced expression of multiple lipogenic genes including sterol regulatory element-binding protein 1c (SREBP-1c), stearoyl-CoA desaturase-1 (SCD-1), and fatty acid synthase (FAS) (31, 64–66). Interestingly, the reduction in lipogenic gene expression arose from the ability of PLA2G10 to generate lipid products that suppress liver X receptor (LXR) activity.

PLA2G10 is expressed in adrenal cells and has a regulatory role in adrenal corticosteroid production. Overexpression of PLA2G10 in C57BL/6 mice resulted in a 30-40% reduction in corticosteroid production, and this effect was reversed by methyl indoxam administration (31). The elevated glucocorticoid level did not arise from elevated ACTH or ACTH responsiveness. PLA2G10 overexpression dramatically reduced expression of the LXR-target gene steroidogenic acute regulatory protein (StAR), a nuclear-encoded mitochondrial protein that mediates the rate-limiting step of steroid synthesis (67). As in the adipocytes, Pla2g10 generated a ligand that reduced LXR activity.

Pla2G10 is also expressed in the pancreatic beta cells and suppresses GSIS (68). Like Pla2g5, Pla2g10 generates AA. However, this pool of AA is converted to prostaglandin E2, which binds to the EP3 receptor. EP3 elevates cAMP leading to decreased insulin secretion. Why Pla2g5 and Pla2g10 have opposite effects on GSIS is unclear. However, Pla2g10 has to be proteolytically activated, and this may give it access to a different pool of phospholipids. Finally, older Pla2g10 KO mice appear to be protected from age-related glucose intolerance.

The current data on PLA2G10 indicate it impacts multiple aspects of metabolism and hormonal action. PLA2G10 expression reduces weight gain and overall adiposity in mice. Concerning hormone actions, Pla2g10 decreases corticosteroid production in adrenal cells (31). Excessive use or production of glucocorticoids will induce insulin resistance, weight gain, and adiposity while also exacerbating Type 2 diabetes (69–72). However, Pla2g10 reduces GSIS, giving this phospholipase a complex contribution to the overall metabolic state.

## PLA2G12B

PLA2G12B is the only phospholipase implicated in metabolism showing no catalytic activity due to a point mutation in its active site, and thus it is hypothesized to act as a ligand for receptors that are currently unidentified (8). Using *Pla2g12b^-/-^* mice fed an *ad libitum* chow diet, knockout of the *Pla2g12b* gene increased TG, cholesterol, and free fatty acids in the liver, resulting in severe hepatosteatosis (35). Hepatocyte nuclear factor-4alpha (HNF-4α) and its co-activator PGC-1α induce *Pla2g12b* expression (35, 36), resulting in the induction of genes involved in lipoprotein packaging (microsomal triglyceride transfer protein, MTP) and VLDL secretion (37, 38). Moreover, liver-specific *HNF-4α^-/-^* mice are phenotypically similar to *Pla2g12b^-/-^* mice, as both lines have reduced serum TG and cholesterol levels and display severe hepatosteatosis (35, 39). These observations suggest that PLA2G12B is one gene involved in the control of lipid metabolism downstream of HNF-4α. Infection of mice with an adenovirus encoding *Pla2g12b* into *Pla2g12b^-/-^* mice improves hepatic VLDL secretion and restores the decline in serum TGs (35). These data indicate that PLA2G12B is under the regulation of HNF-4α and plays a metabolic role by regulating lipoprotein packaging and VLDL secretion in the liver. More recent data has shown that estrogen-related receptor γ (ERRγ) transcriptionally regulates PLA2G12B through binding to its promoter region to regulate hepatic VLDL-TG secretion (73). In *Pla2g12b^-/-^* mice, ERRγ fails to regulate VLDL-TG secretion and large hepatic lipid droplets result. Importantly, ERRγ is implicated in a wide range of physiologic roles including metabolic homeostasis, especially hepatic glucose metabolism (40). The findings associating PLA2G12B with ERRγ may be one avenue by which ERRγ mediates hepatic glucose production.

## Conclusion

**Figure 1:**
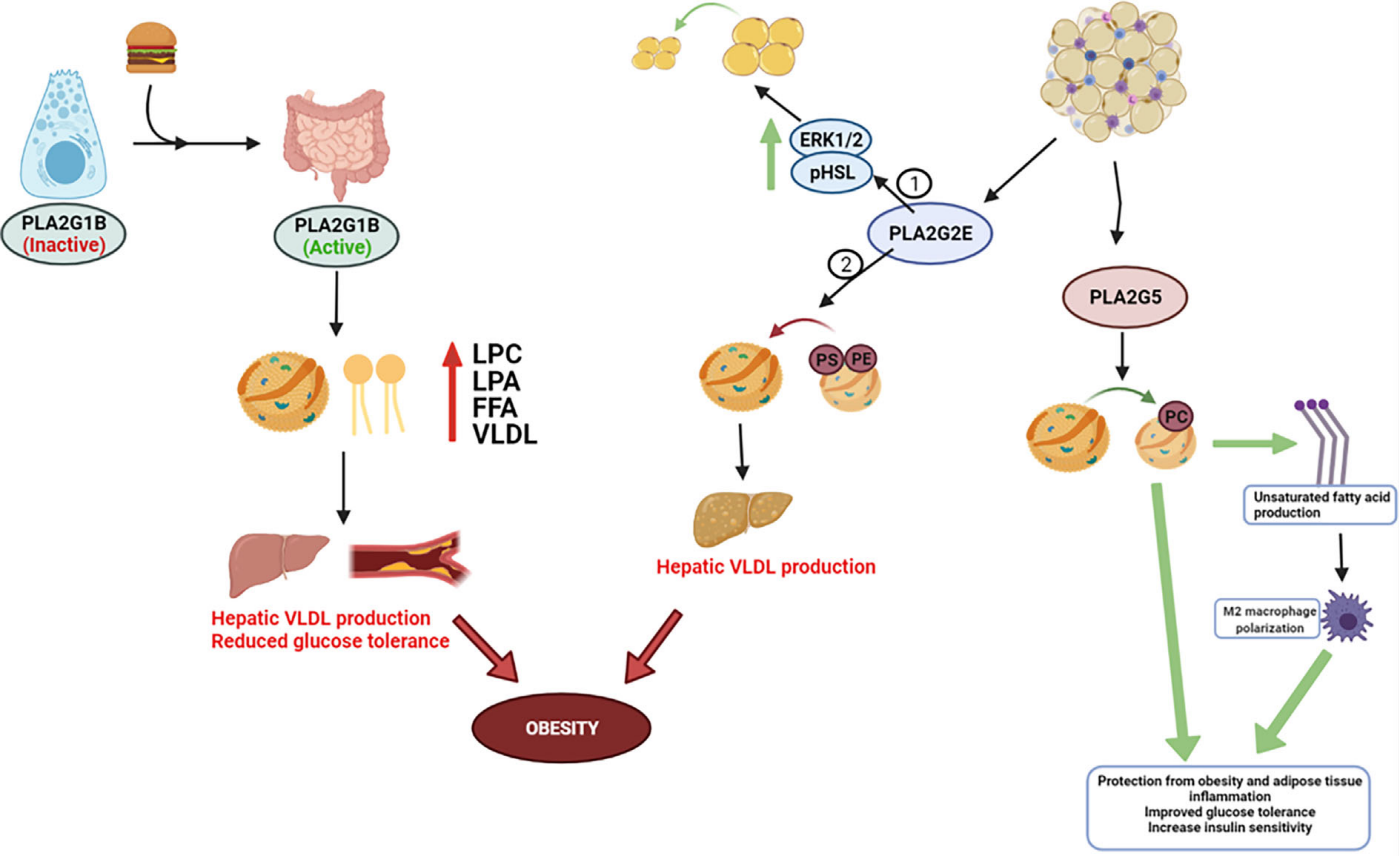
Metabolic role for PLA2s. PLA2G1B is released by pancreatic acinar cells into the pancreatic juice following a meal and then secreted into the intestinal lumen, where it eventually becomes activated. Phospholipid digestion by PLA2G1B results in elevated lysophosphatidylcholine (LPC), lysophosphatidic acid (LPA), and free fatty acids absorption in the portal blood, plasma, and liver, and increases hepatic very-low density lipoprotein (VLDL) production. Elevated enzymatic activity in the intestinal lumen can progress obesity, reduce glucose tolerance, and exacerbate insulin resistance – most notably due to elevated release/absorption of LPC and overarching dyslipidemia. PLA2G2E and PLA2G5 are expressed in WAT. 2 studies have contradicting results in PLA2G2Es metabolic role – 1 study discovered PLA2G2E increases ERK1/2 signaling and HSL phosphorylation to increase lipolysis, whereas Study 2 found PLA2G2E may promote obesity through hepatic lipogenesis and VLDL production. PLA2G5 preferentially hydrolyzes PC-rich phospholipids and promotes M2 macrophage polarization, which appears to have a beneficial impact on LDL lipid normalization and whole-body insulin sensitivity.

The recent studies discussed in this review show that sPLA_2_s can influence metabolic diseases such as Type 2 diabetes and obesity, at least partially through alterations in lipid production and mobilization (**Figure 1**); and while controversy exists regarding whether elevated lipids directly cause insulin resistance, pre-clinical and clinical data indicate an association between elevated lipids and lipoproteins with insulin resistance (74–77). Furthermore, one issue in analyzing the metabolic impact of sPLA2s is many of the isoforms effect intertwined pathologies such as atherosclerosis, heart disease, and cancer (41, 55, 57, 64–67, 69–72). For this reason, future studies on sPLA_2_s should consider their roles based on tissue localization, as their distinct functions may be altered based on the tissue being analyzed. Furthermore, the expression and compensation of other sPLA_2_ isoforms in transgenic animal models is another factor that might result in large phenotypic changes, and thus should also be observed to advance what we know about sPLA_2_s in metabolic diseases.

## Author Contributions

The author confirms being the sole contributor of this work and has approved it for publication.

## Funding

I would like to thank the Wenner-Gren Foundation of Sweden and their post-doctoral fellowship program for the support on this work (UDP2020-0101).

## Conflict of Interest

The author declares that the research was conducted in the absence of any commercial or financial relationships that could be construed as a potential conflict of interest.

## Publisher’s Note

All claims expressed in this article are solely those of the authors and do not necessarily represent those of their affiliated organizations, or those of the publisher, the editors and the reviewers. Any product that may be evaluated in this article, or claim that may be made by its manufacturer, is not guaranteed or endorsed by the publisher.
